# Functional EEG connectivity during competition

**DOI:** 10.1186/s12868-018-0464-6

**Published:** 2018-10-18

**Authors:** Michela Balconi, Maria Elide Vanutelli

**Affiliations:** 1 Research Unit in Affective and Social Neuroscience, Catholic University of the Sacred Heart, Milan, Italy; 2 Department of Psychology, Catholic University of the Sacred Heart, Largo Gemelli 1, 20123 Milan, Italy

**Keywords:** Competition, Hyperscanning, EEG, Functional connectivity, Reinforcing feedback

## Abstract

**Background:**

Social behavior and interactions pervasively shape and influence our lives and relationships. Competition, in particular, has become a core topic in social neuroscience since it stresses the relevance and salience of social comparison processes between the inter-agents that are involved in a common task. The majority of studies, however, investigated such kind of social interaction via one-person individual paradigms, thus not taking into account relevant information concerning interdependent participants’ behavioral and neural responses. In the present study, dyads of volunteers participated in a hyperscanning paradigm and competed in a computerized attention task while their electrophysiological (EEG) activity and performance were monitored and recorded. Behavioral data and inter-brain coupling measures based on EEG frequency data were then computed and compared across different experimental conditions: a control condition (individual task, t0), a first competitive condition (pre-feedback condition, t1), and a second competitive condition following a positive reinforcing feedback (post-feedback condition, t2).

**Results:**

Results showed that during competitive tasks participants’ performance was improved with respect to control condition (reduced response times and error rates), with a further specific improvement after receiving a reinforcing feedback. Concurrently, we observed a reduction of inter-brain functional connectivity (primarily involving bilateral prefrontal areas) for slower EEG frequency bands (delta and theta). Finally, correlation analyses highlighted a significant association between cognitive performance and inter-brain connectivity measures.

**Conclusions:**

The present results may help identifying specific patterns of behavioral and inter-brain coupling measures associated to competition and processing of social reinforcements.

## Background

Social behavior and social interactions pervasively shape and influence our lives and relationships, it is then not surprising that investigation of the so-called “social brain” and of the neural bases of human social skills is attracting more and more attention [1]. Within this scenario, cooperation and competition are the primary (and opposite) interaction dynamics that define different ways to jointly execute a common task.

Previous studies underlined the importance to explore cooperative interactions since, considering mankind social organization, it constitutes a source of positive social feedback. In fact, driven by empathic and prosocial concern, the satisfaction of affiliative, shared needs can often become a social reward per se [2]. Competition, on the other hand, stresses the relevance and salience of social comparison processes between the inter-agents that are involved in the task, and includes other psycho-social issues related, for example, to the adoption of social hierarchies as a landmark. Thus, it is possible to imagine that the behavioral and neural effects corresponding to these two mechanisms are reflected by different and specific cognitive, neural, and behavioral patterns [3]. Few previous works directly compared these two conditions. A previous fMRI study [4] showed that, although the two conditions share some neural correlates related to social cognition, they are anyhow associated with different networks. In detail, cooperative actions seem to recruit orbitofrontal areas, while prefrontal and more posterior (parietal) cortices are involved during competition. The authors interpreted such result starting from evolutionary and developmental psychology and stressed the highly rewarding effect of cooperation and a sort of merging of the two partners. Conversely, competition seems to involve less inclusion and a clear separation between the self and the other. Interestingly, a recent hyperscanning study seems to be in line with such evidence, since it revealed that two cooperative partners show increased behavioral and neural synchrony than competitive ones during a joint task [5]. This result was motivated as a sort of disengagement from the members of the couple, and a similar effect was also observed in the case of inefficient joint interactions [6–9]. Thus, although it is significant to explore cooperation as a highly gratifying, positive, and rewarding condition, the effects related to disengagement, social exclusion, social differentiation and hierarchic mechanisms deserve greater attention.

From an experimental point of view, given the intrinsic complexity of the phenomenon, recent theoretical advances in social neuroscience lead to a change in perspective and underlined the importance of considering interacting agents as inter-dependent parts of a system in order to properly understand social behavior [10, 11]. Nonetheless the majority of studies on social interaction skills is based on “one-person paradigms” where an individual participant perform actions addressed to human or non-human agents, or where two participants are asked to participate in the same task but do actually act just one at a time, following turn-taking rules [4, 12].

For example, previous studies [12, 13] required subjects to perform the task while their performance was compared to that of a peer group. Of course, these competitors did not exist, and specific fake feedbacks were displayed about subjects’ performance compared to the others. In this case, results showed that the social manipulation in terms of both performance and ranking position was able to modulate subjects’ behavioral and neural responses. In detail, a better performance with a left frontal lateralized pattern emerged in the case of a positive and proficient self-perception (win condition), while a worse performance with a right asymmetry was revealed in the lose condition, connoted by negative emotions and poor self-perception.

For what concerns turn-based paradigms, instead, a recent functional near-infrared (fNIRS) study compared cooperative and competitive dynamics during a game. Participants were assigned to two different roles: game builder or partner. Results showed that the builder’s activation in the right inferior frontal gyrus (IFG) was increased or reduced while interacting with a cooperative or competitive partner, respectively.

However, given the turn-based structure of classical investigation paradigms, even when different participants take part in the same experimental procedure, neural activation data related to their thoughts, choices and behaviors refer to different phases of the interaction and then cannot be analyzed to explore proper inter-brain synchronization associated to social interaction dynamics and to simultaneous adaptation of participants’ behaviors.

Consequently, in order to investigate social exchanges between competing agents and related neural activities, we moved towards a “two-person perspective” [14–16] and implemented an hyperscanning paradigm [17, 18], where bodily activities of two interacting agents are simultaneously recoded, matched, and analyzed together. We therefore decided to explore brain-to-brain coupling in terms of functional connectivity, understood as the temporal correlation between neurophysiological events that are spatially remote and measured as simultaneous coupling between two time series of biosignal data collected from different inter-agents. Connectivity analyses based on electroencephalographic (EEG) data have the advantage, over methods based on functional imaging data, of being characterized by higher temporal resolution and, then, of being able to mirror swift modulations of moment-by-moment interactions. Such features makes EEG-based hyperscanning a valuable tool to explore social interaction dynamics, as suggested by the first few evidences in literature concerning different interaction situations [19–21]. This advantage of hyperscanning techniques over conventional paradigms also emerged in a previous study [22] comparing cooperation and competition between a joint condition, where both subjects played together, a solo condition, where both subjects were asked to complete the task individually, and a condition against a PC. The comparison between the joint and PC, as well as between the joint and the individual task, revealed significant differences in terms of inter-brain functional causal relations.

Further, electrophysiological recordings allow for assessing modulation of oscillatory activity associated to cognitive load. For example, Babiloni et al. [23], in a study where participants were asked to play a card game, found larger activity in prefrontal and anterior cingulated cortex across different frequency bands in the player that leaded the game with respect to the other ones. A successive study with the same paradigm [24] integrated such results with functional connectivity analyses and found that the pattern of inter-brain connectivity in the cooperation condition was denser than in the defect one. In fact, the individualistic choice could have produced a lower synchronization between brains. On the other hand, a cooperative act elicited a weaker brain activity, but a denser synchronization between the two brains. In addition, another work by Sinha et al. [25] showed that the competitive condition was characterized by significantly lower synchronization as compared to cooperation.

Consistently, it was observed that competition lead to increased cognitive load and cortico-cortical communication, likely due to higher efforts linked to strategy planning, as mirrored by modulations of alpha frequency power. In fact, a previous experiment revealed decreased left alpha activity (increased brain response) after a competitive reinforce [26].

Strategy planning, in particular, is a critical cognitive skill and a crucial aspect for inter-personal regulation during competition. Relevantly, such skill shapes inter-agents’ actions on the basis of self-perception and attribution of efficacy and of information on one’s own and other’s performance. Moving in that direction, in previous investigations of competition (or cooperation) dynamics, the presence of an external feedback informing participants on their performance—in particular when it is positive—proved to be able to modulate their behavioral responses [17, 26–28]. While it has been suggested that even such modulation may be mediated by dorsolateral prefrontal structures [29], potential effects of processing information conveyed by performance feedbacks on inter-brain neural synchronicity and inter-agents synergies are yet to be explored.

The present study aims at investigating inter-personal synchronization during a competitive task by exploring inter-brain coupling of EEG activities. Further, we will explore the effect on performance and EEG synchronization of receiving an external positive feedback about individual performance. Moreover, being a task involving social and affective components, we were particularly interested in exploring the presence of lateralized patterns to better interpret results at light of subjects’ emotional experience. Going down to specifics, we expected that: (1) participants will do fewer errors and will decrease their reaction times after receiving a positive feedback on their performance as a function of the perception of increased efficacy; (2) inter-brain coupling will decrease as the competitive task goes on—and in particular after receiving the positive feedback on individual performance—following reduced interpersonal engagement and implementation of individual strategies instead of joint action plans; (3) the modulation of inter-brain coupling could be primarily observed in prefrontal areas, given their critical role for higher social skills necessary for inter-personal tasks [12, 13, 30] and, in particular, competition [26].

## Results

Three different steps of analysis were conducted. First, behavioral data were analyzed. Then, EEG connectivity indices were computed. Finally, correlational analyses were run between these two. To avoid the presence of confounding factors such as a learning effect during the task due to the repeated conditions, a preliminary check was performed to compare the first four blocks (1–4) and blocks 5–8 for all the dependent variables of interest (RTs, ERs, EEG). Since the analyses did not reveal significant differences between the two sets, this factor was not further included in the three formal steps.

### ERs and RTs

**Fig. 1:**
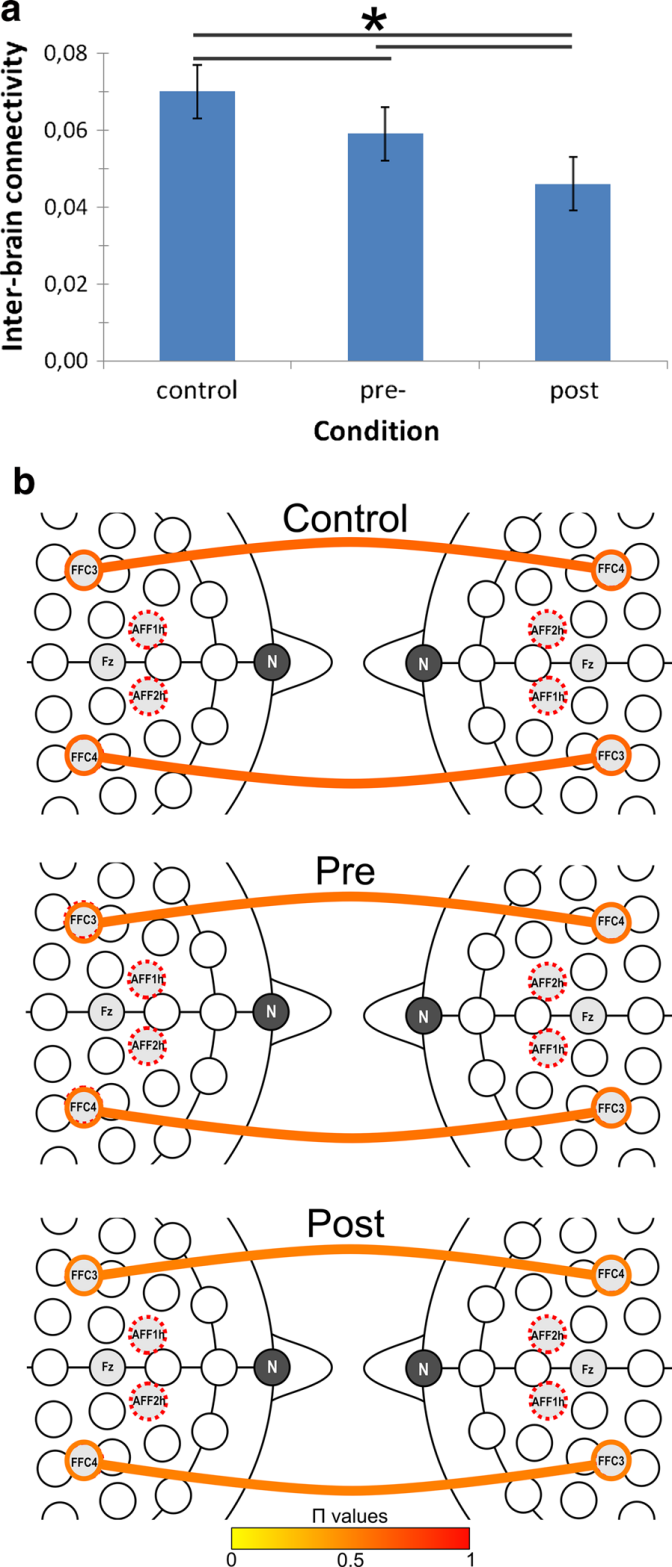
Histograms (**a**) and EEG inter-brain functional connectivity patterns (**b**) as a function of Condition and Localization for the theta band, Π values. Bars represent ± 1 SE around group means. Asterisks mark statistically significant differences (*p* < .05). Colored lines represent the strength of the relation, ranging from 0 (yellow) to 1 (red)

Two repeated measure ANOVAs were performed with ER and RTs dependent measures. The independent factor was Condition (Cond. 3 levels: control; pre; post-feedback). Considering ERs, ANOVA models highlighted the significant effect of Cond factor (*F*[2, 23] = 9.78, ≤ .001, η^2^ = .40), with decreased ERs in post-feedback with respect to pre-feedback sessions (*F*[2, 29] = 9.15, *p *≤ .001, η^2^ = .39). Similarly, ANOVA models highlighted the significant effect of Condition on RTs (*F*[2, 29] = 8.75, *p *≤ .001, η^2^ = .38), with decreased RTs during post-feedback sessions with respect to the control task (*F*[2, 29] = 8.18, *p *≤ .001, η^2^ = .37) and pre-feedback sessions (*F*[2, 29] = 9.05, *p *≤ .001, η^2^ = .39). Pre-feedback RTs were also significantly lower than those collected during the control task (*F*[2, 29] = 7.91, *p *≤ .001, η^2^ = .35) (Fig. 1a, b).

### Inter-brain connectivity

The second set of ANOVA models was applied to inter-brain connectivity data, with Condition (Cond: control; pre; post-feedback), Localization (Loc: 4 levels. AF; F; C; P), and Lateralization (Lat: 2 levels. Left; Right) as fixed factors. Greenhouse–Geisser correction of degrees-of-freedom was applied to ANOVA outcomes when needed. Simple effects for significant interactions were further checked via pair-wise comparisons, and Bonferroni correction was used to reduce multiple comparisons potential biases. Furthermore, the normality of the data distribution was preliminary assessed by checking kurtosis and asymmetry indices.

**Fig. 2:**
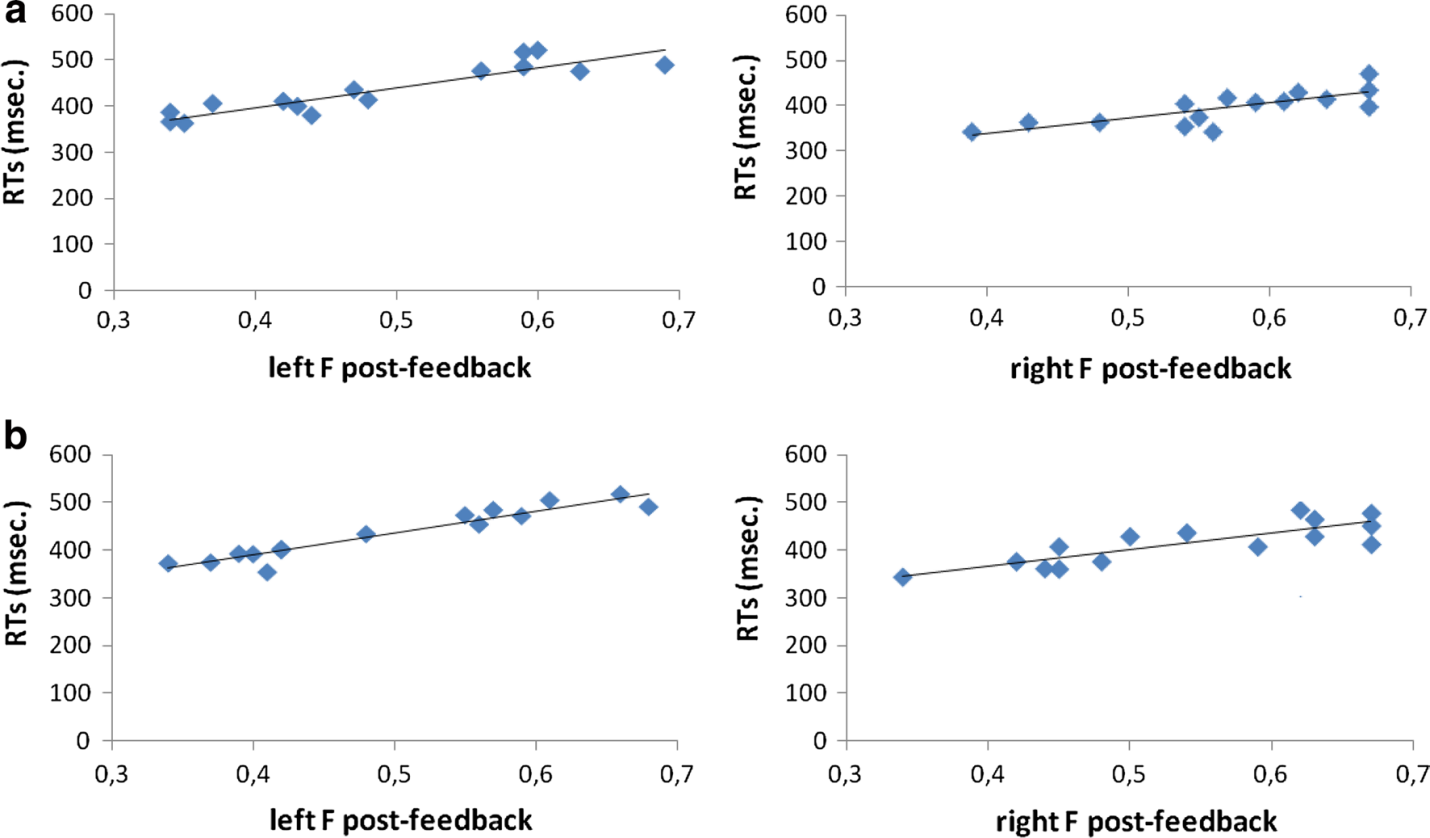
Correlation analyses. RTs revealed significant correlations with inter-brain connectivity measures within right and left prefrontal areas during post-feedback session: (**a**) delta frequency band; (**b**) theta frequency band

As for delta activity, the ANOVA model applied to inter-brain connectivity values showed significant Cond (*F*[2, 28] = 9.12, *p *≤ .001, η^2^ = .39) and Cond × Localization (*F*[6, 82] = 9.11, *p *≤ .001, η^2^ = .38) effects. As for the main effect, lower inter-brain connectivity was observed in post-feedback than pre-feedback (*F*[1, 14] = 8.56, *p *≤ .001, η^2^ = .36) and control (*F*[1, 14] = 8.45, *p *≤ .001, η^2^ = .35) condition. As for the significant interaction effect, pair-wise analyses highlighted that—within F recording channels—inter-brain connectivity decreased during post-feedback with respect to pre-feedback sessions (*F*[1, 14] = 9.77, *p *≤ .001, η^2^ = .39) and control condition (*F*[1, 14] = 10.01, *p *≤ .001, η^2^ = .41). In addition, in F localization, inter-brain connectivity was lower during pre-feedback sessions than during the control condition (*F*[1, 14] = 9.12, *p *≤ .001, η^2^ = .40) (Fig. 2a, b). No other effect was found to be statistically significant.

**Fig. 3:**
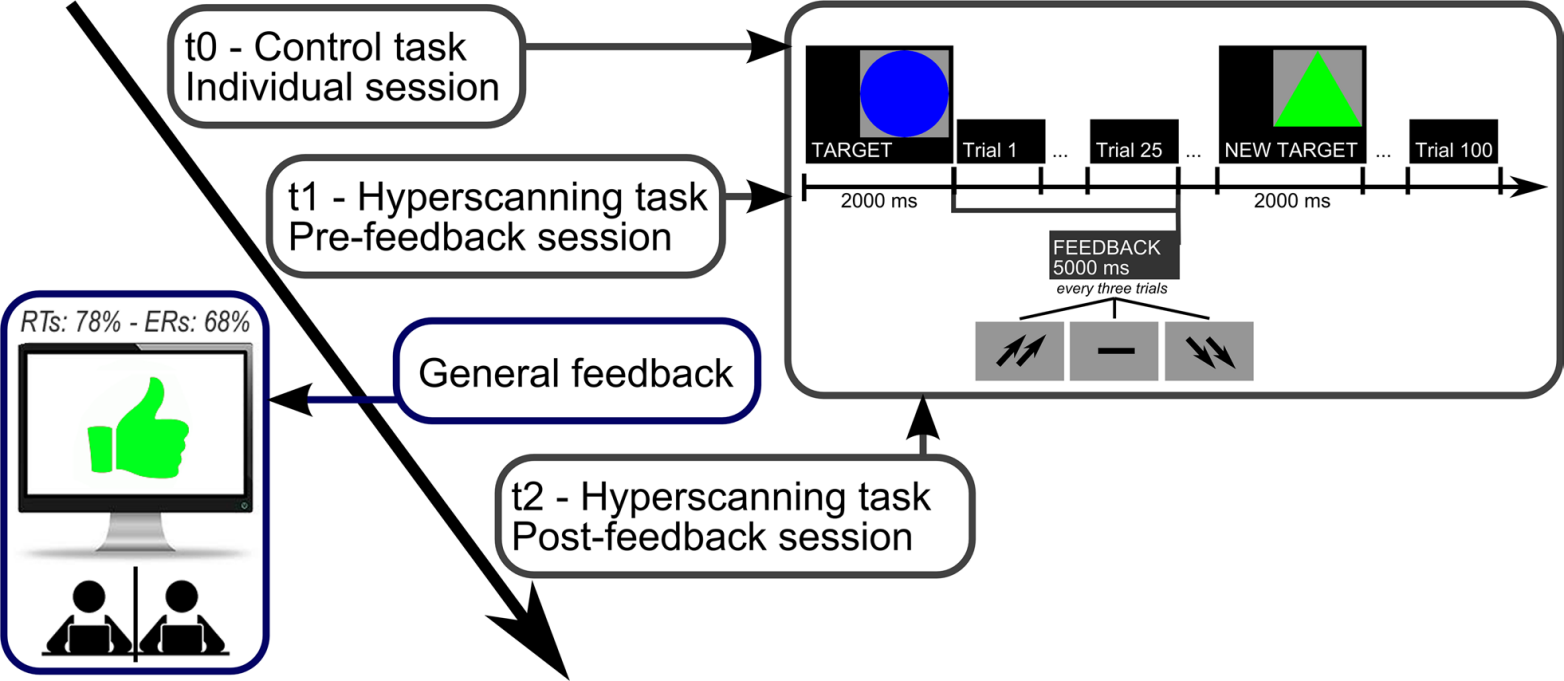
Experimental design and task/trial structures

As for the theta frequency band, significant effects were observed for Cond (*F*[2, 28] = 9.32, *p *≤ .001, η^2^ = .39) and Cond × Localization (*F*[6, 62] = 8.44, *p *≤ .001, η^2^ = .37) effects. As for the significant main effect, lower inter-brain connectivity was observed in post-feedback than pre-feedback condition (*F*[1, 11] = 9.03, *p *≤ .001, η^2^ = .37). Moving to the interaction effect, inter-brain connectivity decreased in post-feedback than in pre-feedback (*F*[1, 14] = 8.16, *p *≤ .001, η^2^ = .36); and control (*F*[1, 14] = 8.70, *p *≤ .001, η^2^ = .37) conditions over F recording channels. Finally, over frontal areas, inter-brain connectivity was lower during pre-feedback than control conditions (*F*[1, 12] = 8.23, *p *≤ .001, η^2^ = .35) (Fig. 3a, b). No other effect was statistically significant.

Alpha and beta bands data did not show statistically significant differences.

### Correlation analysis

Correlation analyses (Pearson correlation coefficients) between behavioral (RTs and ERs) and neurophysiological (inter-brain EEG connectivity) measures were computed in order to investigate potential reciprocal associations across those levels.

**Fig. 4:**
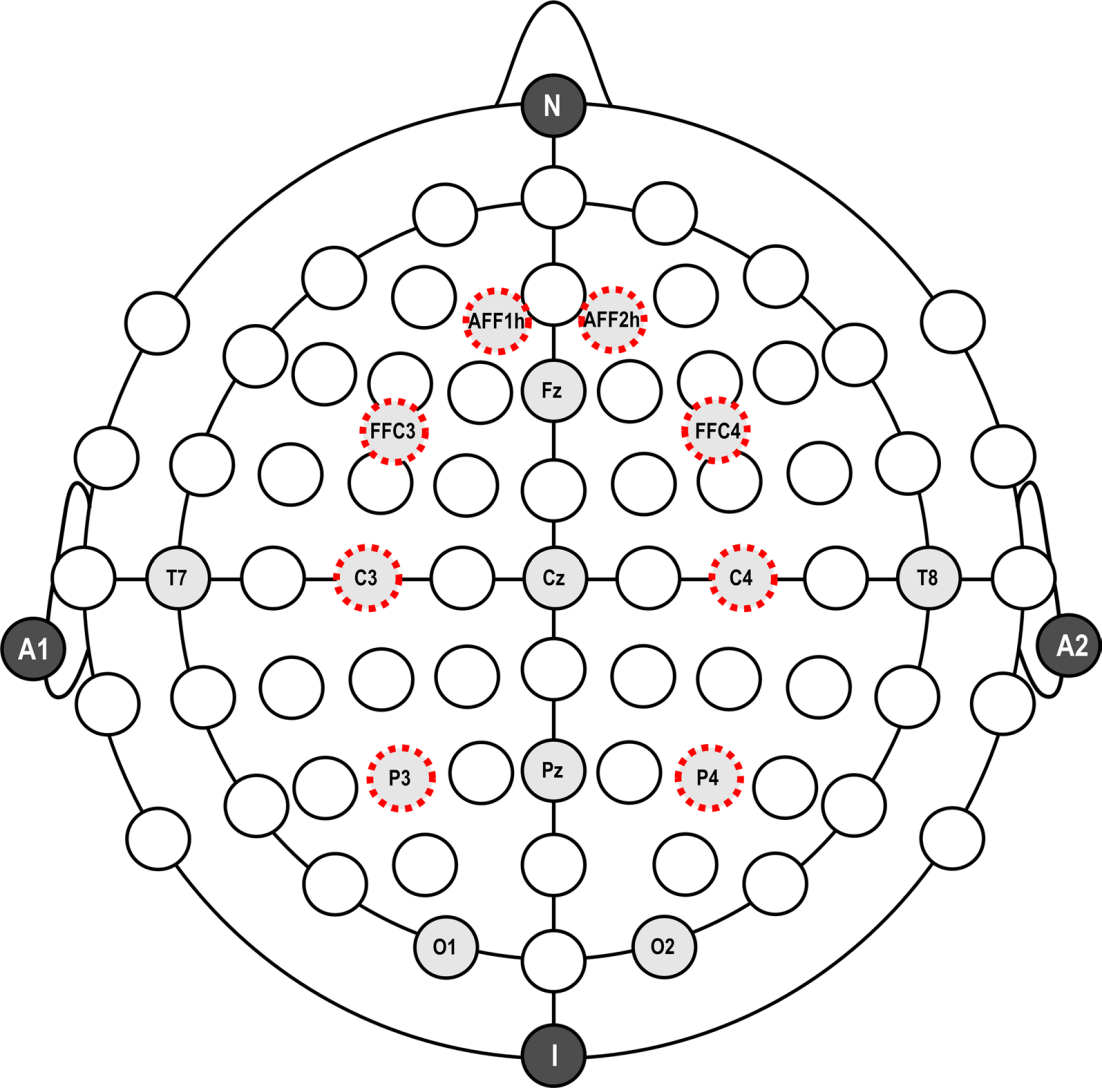
EEG montage. Electrodes located on left and right anterior frontal (AFF1h, AFF2h), frontal (FFC3h, FFC4h), central (C3, C4), and parietal (P3, P4) sites (dashed contour) have been included in connectivity and statistical analyses

As shown by Pearson correlation coefficients, delta band values concerning left and right frontal areas and RTs proved to be positively associated during the post-feedback session (respectively *r* = .543, *p *≤ .001; *r* = -.513, *p *≤ .001). Namely, lower right/left DLPFC connectivity was related to reduced RTs values in post-feedback condition. Similarly, as for theta activity, significant positive correlations were found between RTs and inter-brain connectivity within left and right F localization in post-feedback condition (respectively *r* = .514, *p *≤ .001; *r* = -.498, *p *≤ .001) (Fig. 4a, b). No other association was statistically significant.

## Discussion

The present study explored cognitive and neural correlates of inter-personal synchronization associated to competitive social dynamics by using a hyperscanning approach. Primary findings highlighted: (1) the effect of competition on cognitive performance, with increased performances during competitive with respect to control tasks, and the salience of an external reinforcing feedback concerning performance levels; (2) a downward modulation of inter-brain connectivity associated to competition; and (3) a significant relationship between brain and behavioral measures.

Firstly, competitive situations were found to produce better behavioral performance when compared to individual (control) conditions. We indeed observed decreased RTs and ERs when subjects had to compete. Specifically, as compared to individual task condition (t0), the presence of a clear competitive connotation (t1) leads to better behavioral performance. That main result is in line with previous evidences, which pointed out the role of competitive contexts, when compared to cooperative ones, in inducing improved cognitive outcomes [31]. As suggested by a previous study by Balconi and Vanutelli [26], such phenomenon might be even more evident when we perceive a positive feedback coming from a win situation. However, in that study the absence of a control condition limited the general extent of conclusions about the significance of competition and perceived superiority effects.

The present critical contrast between an individual and a competitive condition may lead to more stringent conclusions on the role of competition in improving cognitive performance. Further, it also helps in interpreting the further increase of cognitive performances after participants received the global reinforcing feedback related to their performance level (t2), which strengthened their self-perception as better performer with respect to their competitor.

Secondly, we even observed a gradual decrease of inter-brain coupling measures related to prefrontal areas moving from control condition to competitive tasks. Such finding may be explained by taking into account the competition frame and the actual task instructions, which clearly defined participants as co-acting competitors and likely lead them to act as individual agents. Indeed, within such frame, even if participants were involved in the same task, participants would have benefit more from individual and self-focused strategies than from joint action plans. In fact, in this case, the neutral condition without a reinforced competitive instruction showed a “baseline” higher connectivity between two persons during a standard joint action. On the contrary, when participants are required to compete, a sort of disengagement of the joint dynamic occurred.

Previous evidence, in fact, underlined that, if compared to cooperative tasks, competition is associated with decreased inter-brain connectivity. In fact, competitive dynamics seem to involve less inclusion mechanisms than cooperative ones, and a clear separation between the self and the other [4]. Cooperation, instead, creates a bond, an overlapping, between the two inter-agents, which leads to increased connectivity patterns [5, 24, 25]. Interestingly, a similar effect was also observed in the case of inefficient joint interactions [6–9].

Moreover, the decrease of inter-brain coupling was particularly evident over bilateral prefrontal areas, and that is in line with both previous EEG-based hyperscanning evidences [32] and literature concerning the involvement of prefrontal structures in the neural network supporting co-regulation of joint actions, strategic planning in social tasks, social exchanges, perspective-taking and mentalization [33–37]. Moreover, an involvement of prefrontal regions already emerged in a previous fMRI study [4] during competitive conditions, while cooperation was more associated with orbitofrontal activity.

While the localization of observed effects is consistent with previous literature on neural signature of social interaction, it is however worth noting that the modulation of inter-brain connectivity associated to our experimental manipulation was present in specific low-frequency components of EEG—namely delta and theta oscillations—suggesting that they might mirror social regulation and emotional engagement processes. Strategic control and conflict monitoring in social situations have indeed been associated to the increase of frontal theta oscillations [38, 39]. Again, the amplitude of alpha/theta bands proved to be correlated to behavioral synchronization of speech rhythms in an hyperscanning EEG investigation of verbal interactions [19], and to mirror even empathy for pain [40]. Furthermore, synchronization of theta and delta oscillations is stronger in response to high-arousal and emotionally-connoted stimuli with respect to neutral ones, and tend to be greater in individuals experiencing deeper emotional engagement [41–43]. We then suggest that the specific modulation of theta and delta activities might be linked to the motivational and attentional value of ongoing social dynamics and to processing of relevant social-affective cues [44–46]. By inducing participants to compete, we indeed created a moderately stressful situation where they engaged with the task, felt to be affectively involved, and enacted individual strategies (instead of synchronized action plans) in order to perform better than their competitor, thus mainly focusing on themselves and reducing inter-personal tuning.

Thirdly, we also observed significant correlations between behavioral performance and inter-brain functional connectivity measures related to prefrontal areas, which were associated with systematic brain-to-brain coupling modulation. Going down to specifics, we noted a systematic convergence of increased cognitive performance and reduction of inter-brain connectivity between the two inter-agents. Thus, while from the one hand we may speak about a general individual “cognitive gain” stimulated by competition and by the presence of the reinforcing feedback, from the other hand this effect occurred at the expenses of the joint dynamics. In fact, competition might have triggered a decreasing trend in inter-brain functional connectivity following induced individualistic strategies. In sum, we may suggest that both behavioral and electrophysiological measures were effective in mirroring the effect of competition and of social reinforcement, and that these levels might similarly offer markers of the impact of external conditions which stress individual instead of inter-subjective goals.

## Conclusion

To conclude, future research might try to get a better sketch of competition dynamics and their correlates by implementing competitive task in even more realistic social contexts able to ingenerate competitive intentions in a more ecological way. Secondly, future research may benefit from wider samples, so to better specify and qualify the brain-to-brain coupling phenomenon even taking into account other potentially relevant mediators (such as gender and some psychological constructs, e.g. empathy and social skills). Moreover, future analyses should better explore the effects related to both positive and negative feedbacks, in order to provide a complete scenario of the competitive dynamics. Finally, considering previous imaging studies revealing specific neural networks for cooperation and competition, further development should also consider a multi-method approach with combined techniques that can provide both temporal and spatial information of the joint interactions.

## Methods

### Participants

Fifteen couples of young volunteers took part in the study (M_age_ = 24.13, SD_age_ = 1.05, 14 women; age range 20–25, identical for women/male). Couples were constituted by same-sex and age-matched participants who were not familiar before meeting at the experimental session. All participants were right-handed and had normal or corrected-to-normal vision. None of them reported history of neurology or psychiatric disorders, and did not showed pathological scores during an additional initial screening procedure [State-Trait Anxiety Inventory—STAI-Y, [47] Beck Depression Inventory—BDI-II, [48]. All participants gave their written informed consent to participate in the research. The study and experimental procedures were conducted in accordance with the Declaration of Helsinki and were preliminarily approved by the Ethics Committee of the Department of Psychology, Catholic University of the Sacred Heart, Milan.

### Procedure

Participants arrived separately in the lab. Then, they were welcomed jointly by two researchers, one each, who drove them to two different experimental locations. In fact, in order to prevent eye contact or other forms of parallel communication, participants were separated by a black panel. They were seated next to each other in a moderately darkened room in front of two PC monitors, one each. During the experiment, each participant was assisted by a researcher for instructions or help. After stressing the competitive connotation of the task, participants were introduced to a selective attention task [13, 26, 28], where they had to detect and respond to target versus non-target stimuli in a sequence of similar stimuli (blue or green circles or triangles). A new target was presented at the beginning of each block. They were required to memorize the target and then to recognize it among other simple geometric figures by making a two-alternative forced-choice with left/right buttons. Each trial was made up of three stimuli, which were shown for 500 ms and separated by a 300- ms Inter-Stimulus Interval (ISI).

**Fig. 5:**
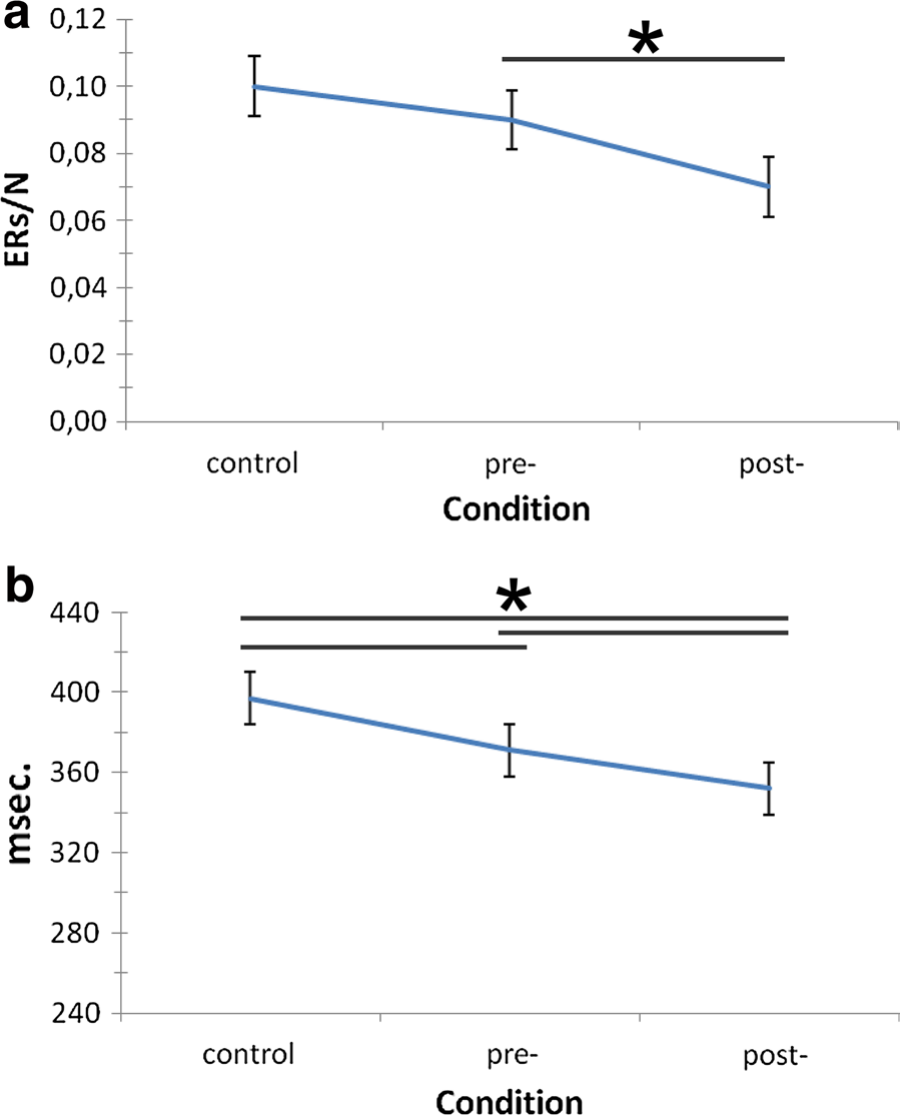
Behavioral results. ERs (**a**) and RTs (**b**) modulation as a function of Condition (control vs. pre-feedback vs. post-feedback). Increased performance was observed in post-feedback with respect to pre-feedback and control conditions. Bars represent ± 1 SE around group means. Asterisks mark statistically significant differences (*p* < .05)

Compared to previous versions of the task, the present version included also a control condition where subjects were not asked to compete, but they were simply required to complete the task by their own (t0; 100 trials). The control condition was then followed by two other experimental sessions (t1 and t2; 100 trials each) where participants had to compete and try to perform at their best. Between the two competition sessions participants received a global feedback concerning their overall individual performance up to that moment. Conditions (control and feedback) were counterbalanced across subjects and presented by a within-subject design. During the tasks, instead, participants received additional real-time feedbacks every three trials: two upward-directed arrows (good trial-specific performance), a dash (mean trial-specific performance); or two downward-directed arrows (bad trial-specific performance). Trial feedbacks lasted for 5000 ms. The EEG activity within this time frame was averaged and used to compute participants’ response to each trial and used to compute synchronization between the two members of the dyad (see also “Connectivity Analysis” section). Then, other 5000 ms occurred as Inter-Trial Interval (ITI). Both trial- and general-feedback were manipulated by the experimenter. As for the between-sessions feedback, all participants were told that their performance was “well above” their competitor’s one and were encouraged to keep the same performance level during the following session (“*Measures recorded till now reveal that your performance is very good. Your response profile is well superior to your competitor’s one*—*about 78% for RTs and 68% for ERs. Keep going like this in the following part*”). As for trial-specific feedbacks, participants received systematic reinforcement across the task by being presented with positive feedbacks in 75% of cases (dash and down-arrows appeared only in 25% of cases and mainly at the beginning of the task so to make the task more credible) (Fig. 5). The experiment lasted about 75 min. Additional information on the task can be found in above-cited published works.

According to qualitative debriefing interviews realized at the end of experimental sessions, participants referred that they were strongly engaged in the competitive task (96%), that they deemed the feedback as veridical (95%), and that their performance at the task was relevant for perceived self-efficacy (97%) as also the perception of having better performances than the other participant (96%).

### Performance data

Reaction times (RTs) were collected from the stimulus onset, and error rates (ERs) were computed as the total number of incorrect target/non-target detections out of the total number of trials (higher values corresponded to increased incorrect responses).

### EEG recording and reduction

Electrophysiological activities were recorded via two EEG systems (V-Amp, Brain Products GmbH, Gilching; Truscan RS, Deymed Diagnostic sro, Hronov) with a 15-channels montage (AFF1h, AFF2h, FFC3h, Fz, FFC4h, C3, Cz, C4, P3, Pz, P4, O1, O2, T7, and T8). Ag/AgCl electrodes were placed according to the 5% International System [49] and referred to earlobes. Sampling rate was set to 500 Hz and electrodes impedance was always kept below 5 kΩ. A 50 Hz notch and a 0.01–250 Hz bandpass were set as input filters. Electrooculogram was collected by placing two additional electrodes above and below the left eye.

EEG data were then analyzed by Vision Analyzer2 Software (Brain Products, Gilching, Germany). Data were filtered offline (0.1-50 Hz bandpass filter, 48 db/oct) and re-referenced to common average, which makes data reference-free.

**Fig. 6:**
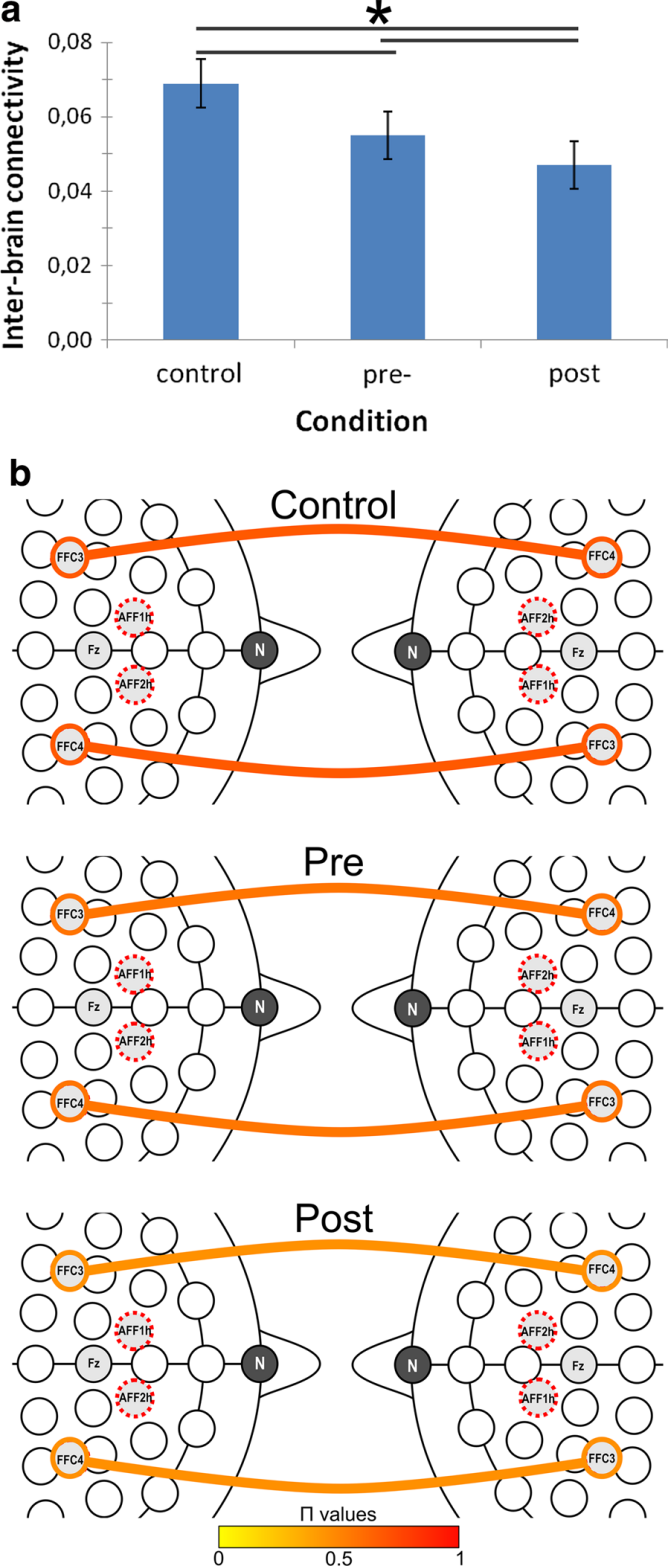
Histograms (**a**) and EEG inter-brain functional connectivity patterns (**b**) as a function of Condition and Localization for the delta frequency band, Π values. Bars represent ± 1 SE around group means. Asterisks mark statistically significant differences (*p* < .05). Colored lines represent the strength of the relation, ranging from 0 (yellow) to 1 (red)

A regression-based ocular correction algorithm suitable for low density montages was applied to data so to reduce artifacts due to saccades and eye-blinks [50]. Signals were then segmented and visually checked so to reject any residual ocular, movement or muscular artifacts. Only artifact-free segments were included in subsequent processing steps. All subjects were included in the analysis since we defined the cut off of 95 trials for each condition. After the visual check, frequency power spectra were computed starting from cleaned waveforms by applying the Fast Fourier Transform. Individual average EEG power values (standard frequency bands: delta—0.5 to 3.5 Hz, theta—4 to 7.5 Hz, alpha—8 to 12.5 Hz, beta—13 to 30 Hz) were finally computed for each recording channel and experimental condition. When performing statistical analyses, we only focused on lateralized activities over anterior frontal—AF (AFF1h, AFF2h), frontal—F (FFC3h, FFC4h), central—C (C3, C4), and parietal—P (P3, P4) areas (Fig. 6). All subjects were included in the final sample, since we defined the cut off of 95 trials for each condition.

### Connectivity analysis

EEG connectivity data were obtained by computing partial correlation coefficients (Π_*ij*_) on subjects’ response to the 300 trial feedbacks (averages of the subsequent 5 s), for each pair of channels, each dyad, and each frequency band. Coefficients were calculated by normalizing the inverse of the covariance matrix Γ = Σ^−1^:

$$\begin{aligned} \varGamma & = \left( {\varGamma_{ij} } \right) = \varSigma^{ - 1} \quad {\text{inverse}}\,{\text{of}}\,{\text{the}}\,{\text{covariance}}\,{\text{matrix}} \\ \varPi ij & = \frac{ - \varGamma ij}{{\sqrt {\varGamma ii\varGamma jj} }}\quad {\text{partial}}\,{\text{correlation}}\,{\text{matrix}} \\ \end{aligned}$$

Correlational analyses have been previously used to assess intra-brain connectivity, especially between frontal areas, with other techniques, such as fNIRS (see for example [51, 52]). In particular, differently from simple correlations, partial correlation quantifies the relationship between two signals (in our case: *i, j*) given (net to) the values of all the other variables that could be directly connected to the model. It is applied in all those cases where the strength of the relationship between two variables is a matter of interest, ranging from computational models [53] to neuroscience. Indeed, the same statistical model was applied in previous work to assess inter-brain synchrony with EEG [54] during failing cooperative interactions. In fact, similarly to the present paradigm, specific averaged values in response to the feedback were used instead of timeseries.

After computing partial correlation values, they were entered into ANOVA models as dependent variables.
